# Lessons from the scale-up of provider-administered and self-injection of DMPA-SC in Nigeria: a landscape assessment

**DOI:** 10.1186/s12905-025-03938-2

**Published:** 2025-09-05

**Authors:** Adewole A. Adefalu, Tolulope O. Afolaranmi, Ebony Fontenot, Rachel Simon, Miranda Buba, Victor Dafe, Funmilayo Olabode, Mary Ajayi, Olajimi Latunji, Joy M. Dogo

**Affiliations:** 1 John Snow Research and Training Institute Inc, Washington DC, USA; 2 Department of Community Medicine, University of Jos, P. M. B. 2084, Jos, Plateau State Nigeria

**Keywords:** Subcutaneous Depot Medroxyprogesterone Acetate, Family Planning services, Nigeria, Contraception

## Abstract

**Background:**

Nigeria is the seventh-most populous country in the world. Its high fertility rate and unmet need for family planning contribute to the increasing population size. To reduce this gap, the Federal Government of Nigeria, in collaboration with Injectables Access Collaborative and other public and private sector players, introduced the subcutaneous depot medroxyprogesterone acetate (DMPA-SC) to the contraceptive method mix in 2017. We conducted an assessment to document the implementation experiences and best practices from the introduction of provider-administered and self-injection (SI) of DMPA-SC from the perspective of the government and implementing partners across states and federal governments levels from the supply-side. This is meant to serve as a learning resource to provide empirical evidence to help inform the DMPA-SC programming.

**Methods:**

A cross-sectional study design that employed a mixed-method approach to data collection was used for this assessment. Our methods included desk review of existing DMPA-SC implementation documents and key informant interviews conducted on 13 government and implementing partners across states and federal levels. Narrative summarization and graphical trend analysis were used for the presentation of information obtained from document review while Nvivo software (version 12) was used for the analysis of transcripts of the KIIs.

**Results:**

This assessment revealed a positive trend in the use of provider-administered and self-injection DMPA-SC from 2020 to 2023. This was supported by functional policies and guidelines. Implementing the Task Shifting and Task Sharing policy continuously; strengthening referral systems for provider-administered and self-injection DMPA-SC programme; and decentralising family planning services were suggested mechanisms for strengthening DMPA-SC programme implementation in Nigeria. However, funding gaps, human resource constraints, and weak coordination mechanisms impeded DMPA-SC implementation scale up.

**Conclusion:**

This assessment illustrates the influence of an enabling environment and stakeholder commitment on the positive trend in provider-administered and self-injection DMPA-SC uptake in Nigeria. Training service providers and improving funding through the use of innovative financing were recommended as levers for DMPA-SC programme sustainability and service scale-up.

## Background

Nigeria is one of the most populous countries in the world, growing at a rate of 3.2% per year and with a doubling time of approximately 27 years [1]. It is the seventh-most populous country in the world, with her high fertility rate and unmet need for family planning being largely responsible for the increasing population size [1]. As of 2019, of the 45 million women of reproductive age (15–49 years) in Nigeria, about 15.7 million (35%) would like to avoid getting pregnant for at least two years, but only 6.2 million (14%) use a modern method of contraception [2]. This leaves about 9.5 million Nigerian women with an unmet need for modern contraception [2].

To narrow this gap, in 2017, the Federal Government of Nigeria, in collaboration with public and private sector partners, made efforts to expand access to modern contraceptives by recommitting to financing family planning to achieve a modern contraceptive prevalence rate of 27% among women by 2024 from 12% in 2018 [3, 4]. One of the strategies for achieving this goal was the introduction of the innovative subcutaneous depot medroxyprogesterone acetate (DMPA-SC) to the contraceptive method mix via social marketing in the private sector and community-based distribution in the public sector [5]. Introduction of DMPA-SC in Nigeria sailed through a milestone of events leading to the current state of implementation. In 2011, the National Agency for Food and Drug Administration and Control (NAFDAC) approved the registration of DMPA-SC-branded products for provider administration. This was followed by approval for DMPA-SC label in 2016 to include self-injection with all the necessary information. This action to a large extent raised awareness of and led to the official introduction of provider-administered and self-injection DMPA-SC in the country [6, 7]. From its introduction in 2016 through the end of 2017, over 1.3 million units of provider-administered and self-injection DMPA-SC were distributed across all states in Nigeria. Between January 2018 and January 2023, 41,566 providers were trained to counsel on and administer it [6, 7]. Various stakeholders have made concerted efforts to improve its availability and accessibility to women, particularly in remote communities, by implementing the Task-Shifting and Task-Sharing (TSTS) policy at the primary health care level [8, 9]. This was done through informed choice counselling to reach additional users and led to an increase in family planning service use, especially of provider-administered and self-injection DMPA-SC. The primary goal of the task shifting and sharing health policy for the country was to support the attainment of universal health coverage and the health needs of the Nigerian population through the mobilization of available human resources to ensure equity, accessibility, and effectiveness in the delivery of essential health care services [9].

In support of these efforts, the Injectables Access Collaborative (AC) is an initiative that is driving increased access to provider-administered and self-injection DMPA-SC as part of an expanded range of contraceptive methods to women and girls focusing on informed choice programming through data-driven technical assistance, coordination, mobilization of resources, and tools. To sustain the gains achieved in provider-administered and self-injection DMPA-SC implementation and scale up, it became important to assess the progress made towards achieving predetermined goals and how the country has been filling implementation gaps, and documenting the lessons and best practices from the process. Many factors influence the uptake of provider-administered and self-injection DMPA-SC and can be categorised into either potential predictors or barriers [10]. By examining these factors, we can understand some of the supply- and demand-side drivers influencing the uptake of this contraceptive method and build a coordinated approach to sustain gains of the introduction. In addition, evaluating the various roles of key actors and stakeholders in provider -administered and self-injection DMPA-SC implementation landscape at the supply side will indicate where more efforts are needed to produce better outcomes and generate empirical evidence to help inform the DMPA-SC programming. It was against this backdrop that we conducted this assessment to document the implementation experiences and best practices from the introduction of provider-administered and self-injection (SI) of DMPA-SC from the perspective of the government and implementing partners across states and federal levels from the supply-side. This is meant to serve as a learning resource to provide empirical evidence to help inform the DMPA-SC programme sustainability and scale-up.

## Methods

### Study area

This assessment was conducted in Nigeria, a country with an estimated population of 211,400,704 at the end of 2021, of which about 49% resides in rural communities [11, 12]. Administratively, Nigeria operates a three-tiered federal system of governance comprising the federal, the 36 states, and the Federal Capital Territory, and 774 local government areas (LGAs). The LGAs are divided into 9,565 political wards, which are the focus of primary health care revitalization to achieve universal health coverage [13]. The modern contraceptive use for all women ages 15–49 years in Nigeria is 12%, while for married women ages 15–49 years is 13% [3, 14]. The injectables method share of the modern method mix is 23% [3, 14]. As a result of access to modern contraceptive use in Nigeria, 2.3 million unintended pregnancies have been averted since 2012 [3, 14]. The unmet need for family planning among sexually active unmarried women is 48% and 19% for those who are married [15, 16]. This implies that the use of modern contraceptive methods in a populous country like Nigeria is abysmally low and requires that all stakeholders make concerted efforts to make the needed change. Among its efforts to achieve the 27% modern contraceptive prevalence rate, [15–17] the Government of Nigeria developed the 2017–2020 national communication plan for family planning; the national guideline and training manuals for the introduction and scale-up of DMPA-SC self-injection; a manual for training of doctors, nurses/midwives, and community health extension workers on postpartum family planning; the TSTS policy for essential health care services; and the standard of practice for family planning [16, 17]. Additionally, the Federal Ministry of Health championed the development of a strategic plan for provider-administered and self-injection DMPA-SC introduction and scale-up to expand access and fast-track progress of Nigeria’s Family Planning Costed Implementation Plan [16, 17].

### Study design

This was a cross-sectional study conducted between April and July 2023 using a mixed-methods approach of data collection to assess the implementation of provider-administered and self-injection DMPA-SC programme. The assessment methods included the desk review of implementation and policy documents; an abstraction of information from the AC dashboard; and key informant interviews (KIIs) with DMPA-SC implementation stakeholders. This methodological approach was employed as it stands to give the needed information for the assessment since the focus of the assessment was on the public sector actors and key programme implementers to gain insight into their learnings particularly on the supply and policy aspect of the DMPA-SC programme implementation. Additionally, we conducted an extensive literature search for articles on provider-administered and self-injection DMPA-SC to support the discussion. The qualitative method of data collection (KIIs) as employed in this assessment offered an opportunity to understanding the implementers’ and policy makers’ perspectives, experiences, and narratives on DMPA-SC implementation through in-depth exploration and analysis.

### Study participants and sampling

Study participants comprised consenting government and implementing partners across states and federal levels. They formed the sampling frame from which a purposive selection was made based on identified roles, responsibilities, availability, and willingness to provide information relevant for the assessment [18, 19]. The number of stakeholders interviewed was influenced by the information saturation principle, such that a point was reached where no new relevant information was obtained from subsequent interviews [20, 21]. In this assessment, the saturation point was reached after the 13 interviews in which responses to the KII questions was not generating any new information.

### Data collection

The desk review involved government policy documents sourced from the Nigeria Federal Ministry of Health, partners implementing provider-administered and self-injection DMPA-SC and family planning services, resources from the AC dashboard, and published research articles. AC dashboard provided triangulated data from the national electronic logistic management system (e-LMIS) and the health management information system (HMIS), including the private sector DMPA-SC uptake data. Information about countries’ DMPA-SC introduction plans and implementation progress was also accessible on the dashboard. The data abstraction tool and KII guide were developed to meet the peculiarities of the assessment, guided by some predetermined questions such as: What are the gaps in the course provider-administered and self-injection DMPA-SC programme implementation? What progress has been made towards achieving provider-administered and self-injection DMPA-SC programme goals? What are the available funding sources and the trends of provider-administered and self-injection DMPA-SC along the scale-up pathway? What are the government’s priorities and directions for sustainability? It also asked questions related to lessons and best practices from implementation. All the existing document with relevant information for answering the afore stated questions were included for the review.

The desk review utilized 6 major existing DMPA-SC implementation and policy documents such as Nigeria Family Planning Costed implementation plan, National Demographic and Health Survey (NDHS), Nigeria TSTS, Standard of Practice for Family Planning (SOP), Strategic Plan for Provider-Administer and Self-Injection DMPA-SC, Nigeria’s Emergency Medicine list.

Data extraction from the Access Collaborative dashboard focused on number of DMPA-SC visits for self-injection over a 3 year period and KIIs conducted on 13 government and implementing partners across states and federal levels who held key strategic DMPA-SC programme implementation positions.

The KII guide was validated by actors and policy makers in the provider-administered and self-injection DMPA-SC implementation environment who were not part of the assessment. A total of 13 KIIs were conducted by the lead researcher and a research fellow, who are public health physicians, via phone and recorded concomitantly. Respondents were contacted at least a week before the interviews for introductions and information about and what to expect. Additionally, verbal informed consent for the interviews and audio-recording was obtained from all participants before the interviews, which lasted 45–60 min. The KIIs were conducted in English Language as all the participants could speak English language fluently.

### Data analysis

All audio recordings were transcribed verbatim into text by two people skilled in transcription. These were supplemented by the KII notes and cross-checked for synergy and completeness by a third person, after which the transcripts were harmonised in preparation for analysis. Thereafter, data familiarization process encompassed reading and re-reading the transcripts to gain insight and understanding of the data and the sequence of expression qualitative research team members. The transcripts were imported into Nvivo software (version 12) for data management, and data concepts, themes, and patterns were identified using the open coding process. This involved labelling sections of the data with codes to identify the key concepts. Next, axial coding was applied to organize concepts and themes into categories. This helped to identify the relationships between the different themes and concepts [22, 23]. The core themes and concepts relevant to the aim of the assessment were then identified using selective coding. Subsequently, content was analysed to evoke a priori and emergent codes, identify recurrent themes, and summarise findings in line with the aim of the assessment and present in narrative formats while word trees and cloud were generated. Review of documents was done by two persons guided by some predetermined questions set to meet the peculiarities of the assessment followed by the development of narrative summarization of the synthesized text and was subsequently verified and collated by a third person into a single document. For the information obtained on the Access Collaborative dashboard which focused on number of DMPA-SC visits for self-injection over a 3-year period from November 2020 to September 2023, a graphical trend analysis was employed for its presentation. Characteristics of the KII respondents such as sex and job description were collated and expressed as text in the narration of the results.

## Results

Of the13 KIIs, six (46%) were women. Respondent job descriptions included executive, clinical, and technical directors; monitoring and evaluation officers; programme managers, family planning coordinators; and reproductive and family planning team leads. See Table 1.

**Table 1:** Characteristics of the KII respondents

Characteristics	Frequency (*n* = 13)	Percentage
Sex		
Male	7	54.0
Female	6	46.0
Job Description		
Executive Director	1	7.7
Clinical Director	2	15.3
Technical Director	1	7.7
Director Family Planning	2	15.3
Monitoring and Evaluation officer	1	7.7
Programme Manager		
Family Planning Coordinator	1	7.7
Reproductive and Family Planning	2	15.3
Team Lead	1	7.7
Programme Officer	2	15.3

### Trends of DMPA-SC implementation in Nigeria

**Fig. 1:**
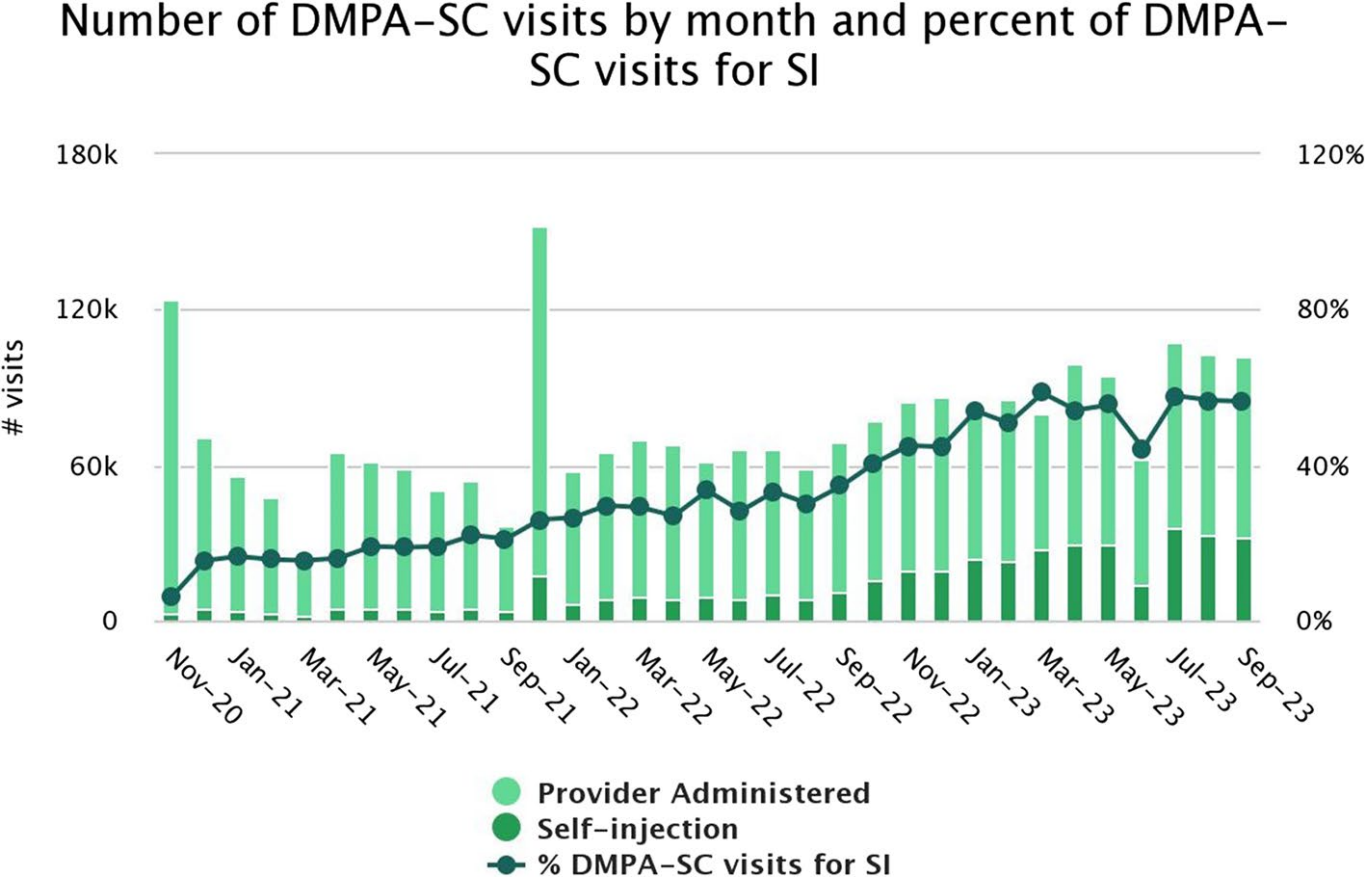
Distribution of DMPA-SC visits for Self-Injection (SI)

Data obtained from the AC dashboard revealed that there has been a steady increase in the use of DMPA-SC as part of the family planning basket of products, either through self-injection by women or provider administration routes. Figure 1 shows a steady increase in the number of DMPA-SC visits for self-injection by month from November 2020 to September 2023 though with a slight dip in May 2023. Furthermore, information synthesized from the desk review revealed that before this landscape assessment, some in-country family planning policy guidelines and initiatives were instituted upon approval and registration of DMPA-SC-branded product by National Agency for Food and Drug Administration and Control (NAFDAC) for provider administration in 2011, which led to the legal use of DMPA-SC. This was closely followed by the approval of the DMPA-SC updated label to include an indication for self-injection in 2016. Additionally, in 2017, the national provider DMPA-SC accelerated introduction and scale-up plan provided a platform for coordinating the activities of various stakeholders. Also, the national guidelines for the introduction and scale-up of DMPA-SC self-injection, which guided the implementation of the DMPA-SC self-injection intervention, was developed between October 2018 and January 2019. Another notable government initiative in family planning was the revision of TSTS policy in 2019, which allowed the expansion of the provider cadre that can administer injectables, including DMPA-SC. Additionally, the inclusion of the provider-administered and self-injection DMPA-SC in Nigeria’s Emergency Medicine List in 2019 as well as the development of the Family Planning Costed Implementation Plan 2019–2023 were also found to facilitate the integration of DMPA-SC into broader family planning programming [19]. Additionally, findings from the KIIs revealed themes that were critical to the increased use of provider-administered and self-injection DMPA-SC as summarized in Table 2.

**Table 2:** Summary of KII findings

Themes critical to improving DMPA-SC use	Gaps in DMPA-SC programme implementation	Efforts to fill DMPA-SC implementation gaps
Evolution in women’s contraceptive choices	Funding, policy, and implementation	Revision and implementation of TSTS policy in 2019
Expansion in the basket of family planning methods	Inadequate human resource and capacity	Policy synergy
Improved acceptance by women	Distribution and logistics gridlock	Policy review and revision
Evidence-driven policy development and coordination efforts	Policy and implementation challenges	Strengthening referral systems and community mobilization
Significant funding support rollout		Sustained advocacy to relevant policymakers

The themes highlighted below were identified in this assessment as being instrumental to the increased use of provider-administered and self-injection DMPA-SC.

#### Evolution and expansion of family planning methods

The utilization of contraceptive methods has significantly evolved over the years in Nigeria, with new products like DMPA-SC and hormonal intrauterine devices (IUD) gaining prominence. This expansion in family planning methods demonstrates the steady improvement and uptake of reproductive health care options, indicating improvements in the availability and quality of family planning services. The uptake of provider-administered and self-injection DMPA-SC as a contraceptive method was expressed to have evolved significantly over the years through the expansion in the basket of family planning methods in Nigeria. This is supported by statements such as “*Over the years, there has been an increase and expansion in the family planning methods for the country*” (Programme Manager). This was reinforced by “*We have seen a lot of new products coming into the country, including DMPA-SC and the hormonal intrauterine device (IUD)”* (Programme Manager). Respondents also highlighted the introduction of provider-administered and self-injection DMPA-SC and the hormonal IUD as two new methods of contraception gaining acceptance in the country. One respondent noted that *“Family planning has really evolved in a positive way, in so many respects*” (Sate Reproductive Health Coordinator)*.* This further illustrates the steady improvement and spread of reproductive health care options in the country over time which could also be a pointer to the fact that there have been improvements in both the availability and quality of family planning services.

#### Improved acceptance and uptake of family planning

The acceptance and uptake of family planning in Nigeria has significantly improved, particularly in northern Nigeria where it was initially low. The introduction of provider-administered and self-injection DMPA-SC and hormonal intrauterine devices (IUD) has led to increased uptake of family planning services. Additionally, task shifting policy, which allows providers to train women in self-injection and make it accessible at the community level, has also contributed to this improvement which has been found helpful in increasing child spacing and reducing family size in the country. Provider-administered and self-injection DMPA-SC including hormonal intrauterine device (IUD) have contributed to the reported improved acceptance and uptake of family planning in Nigeria. This is attested to by the following statements: *"The family planning program in Nigeria has improved a lot"* (Programme Director), and *"There's more uptake of family planning generally around the country"* (Programme Director), indicating that there have been substantial positive changes in the family planning programme in Nigeria with increased uptake of family planning services in Nigeria. Furthermore, statements such as *"Family planning is now more accepted among people, especially in northern Nigeria where initially it was rejected"* (Sate Reproductive Health Coordinator), shows how family planning has overcome cultural and religious barriers to become more widely accepted, especially in northern Nigeria. It is evident that people's views on family planning are changing, and while this may not be directly attributable to provider-administered and self-injection DMPA-SC solely, the product has contributed. *"DMPA-SC is a new method that has gained popularity in recent years"* (Family Planning Coordinator), and *"The journey of DMPA-SC implementation has been interesting, including the introduction of self-injection."* (Clinical Director). These statements reflect that the introduction and acceptance of provider-administered and self-injection DMPA-SC as a reliable and convenient method of contraception is a step in the right direction providing individuals with a reliable and convenient method of contraception. The statement *"The task shifting policy allows providers to train women in self-injection and make it readily available at the community level”* (Project Director). This response indicates that TSTS policy facilitates health system improvement and access because it allowed more cadres of health care workers to train women to inject themselves, promoting the method’s uptake and helping the country increase child spacing and reduce family size.

### Gaps in DMPA-SC programme implementation

The implementation of the national scale-up of provider-administered and self-injection DMPA-SC programme in Nigeria has many challenges, as follows.

#### Funding

The implementation of provider-administered and self-injection DMPA-SC programme in Nigeria faces several challenges. The main issue is inadequate funding, which is affecting the procurement of commodities like DMPA-SC. This has limited the potential of this method to address unmet family planning needs for women of the reproductive age group in the country. The lack of sufficient financial resources is also a major problem for capacity building of key actors and DMPA-SC programme implementation as a whole.

Results from common responses identified inadequate funding as a major gap, as expressed by these statements *"The funding is a major gap"* (Project Director)*,"Funding is affecting the procurement of commodities, including DMPA-SC* (Project Director)*","Funding constraints are a major issue we have"* and *"Challenge with procuring the required quantities of DMPA-SC is enormous"* (Project Director). This further reiterates respondents’ concerns that lack of sufficient funding is a significant challenge in the national scale-up of DMPA-SC/and self-injection implementation. Funding limitations, particularly for procurement of commodities, including DMPA-SC, has impacted negatively on the scale up, limiting the potential the method has to address the unmet family planning needs of the women of the reproductive age group in the country. This was reiterated by this response *"We have a funding gap of about 39 million USD"* (Project Director)*.* This highlights the fact that a substantial funding shortfall exist for procurement of FP methods. This needs to be addressed to adequately support the scale-up efforts of DMPA-SC and other family planning commodities. Furthermore, statements such as *"Getting resources to roll out fast due to financial resources required”*(Project Director), highlights the need for sufficient financial resources to facilitate the rapid and effective rollout of the DMPA-SC implementation and *"Funding is a major problem for capacity building and implementation"* (Project Director) This statement alludes to the fact that adequate financial resources are required to conduct comprehensive and effective capacity-building activities for healthcare providers, program managers, and other stakeholders involved in the DMPA-SC implementation process.

#### Human resource and capacity

Inadequacy of human resource and capacity of service providers in terms of distribution and intuition skill were identified as gaps in the course of scale up of DMPA-SC self-injection programme. Furthermore, capacity of the providers to disseminate context specific messages and addressing social, cultural, and gender norms related to DMPA-SC and other family planning methods requires improvement as expressed by the participants: *"There is inadequate availability of skilled manpower to provide family planning services"* (Project Director), and *"Challenges exist in disseminating messages and addressing social, cultural, and gender norms"* (Executive Director)*.* These statements highlight the challenge of inadequate availability of skilled manpower to deliver family planning services including DMPA-SC. Additionally, responses such as *“Inadequate availability of skilled manpower”* and *“Challenge in getting the aggregate number of forecasts personnel required to provide family planning services exists”* (Male Director), buttresses the challenge with getting the critical mass of trained healthcare personnel needed to support the scale-up of DMPA-SC. Also, statements such as *“Gap in the number of health facilities, especially in difficult-to-reach areas”* (Project Director) *and “Lack of capacity in programme managers in some states”* (Project Director), *highlight* the issue of unequal distribution of health facilities, particularly in remote or hard-to-reach areas, as well as lack of capacity among program managers responsible for overseeing the implementation of DMPA-SC services in certain states. Effective implementation requires skilled manpower that can navigate and address these sensitive and complex issues. The success of the programme relies heavily on an adequate number of skilled healthcare providers trained in family planning services, including DMPA-SC at all levels.

#### Distribution and logistics gridlock

Distribution and logistics gridlock are a major challenge to achieving the desired scale-up of DMPA-SC implementation and other family planning services in the country. Uninterrupted supplies of DMPA-SC and other family planning commodities is hindered by poor logistic systems contributing significantly to reported stockouts and rationing of commodities with resultant disequilibrium between demand with supply. Uninterrupted supplies of DMPA-SC and other family planning commodities is essential for achieving the desired scale up of family planning services. Implementation of scale up of new products may be hampered by poor logistic systems. This is supported by statements such as: *“Last-mile distribution of family planning products, including DMPA-SC, is still a challenge”* (Project Director), as well as *“Stock-outs and rationing of commodities is a challenge”* These statements highlight the difficulty in ensuring the efficient distribution of family planning products, including DMPA-SC to the last mile. Where stockouts exists, it becomes difficult to match demand and supply of DMPA-SC and other family planning commodities. A participant brought to light the need to prioritize both commodities and consumables: “*Gap in ensuring consumables for DMPA-SC are readily available exists”* (Project Director)*.* This assertion unearths the poor understanding of the unique packaging of DMPA-SC; which comes as a prefilled, autodestruct Uniject and requires no additional consumables like syringe, needles as required for other injectable methods like intramuscular form of DMPA or noristerat injection.

#### Policy implementation

The implementation and coordination of provider-administered and self-injection DMPA-SC and other family planning services in the country are challenged with issues of policy operationalization and limited acceptance of policies like TSTS by service providers. Although policies and guidelines are supporting the scale-up of provider-administered and self-injection DMPA-SC and other family planning services in the country, their implementation and coordination are poor. One respondent said that *“These policies don't speak to each other"(M&E Officer),* suggesting poor alignment and streamlining of the policies and guidelines. Furthermore, statements such as *“Challenges in policy operationalization exist in some states"* (Executive Director) denote difficulties in translating policies into action. Additionally, limited acceptance of policies such as TSTS by service providers has hindered DMPA-SC implementation and other family planning services. This was flagged by respondents who noted that *“non-acceptance and use of the TSTS policy is a threat”* (Project Director), and *“Lack of confidence of providers and fear of losing relevance”* (Technical Lead)*.* Providers are worried about losing autonomy and relevance, particularly when family planning tasks are shifted to women themselves. Their concern about being unnecessary if the TSTS policy is fully implemented threatens the scale up of DMPA-SC and other family planning methods.

#### Coordination mechanisms

Poor coordination mechanisms have been identified as a major impediment to the scaling up provider-administered and self-injection DMPA-SC family planning services. Critical examination and improvement in the coordination structures between relevant government agencies is key to improving the efficiency of DMPA-SC implementation in the country. This was reiterated by findings from the study *“Challenges with coordination between key government agencies exists*” (Project Director)*,* This statement underlines the importance of synergy between government agencies in the family planning space for effective programme implementation. It may be necessary to examine the coordination structures that allow interaction between key government agencies and those involved in the implementation of the provider-administered and self-injection DMPA-SC to improve scale-up effort efficiency.

### Efforts to close provider-administered and self-injection DMPA-SC implementation gaps

Five major themes related to DMPA-SC implementation gaps emerged and are described below.

#### Implementation of task shifting and task sharing policy

The implementation of task shifting and task sharing policies is central to addressing the policy gaps in the DMPA-SC scale-up in the country with emphasis on targeted implementation based on demands for DMPA-SC services. Furthermore, the collaborative efforts of partners at the subnational level are vital to achieving optimal TSTS policy implementation. Results identified the theme addressing of task shifting and task sharing policy implementation to address policy gaps. This is attested to by statements such as: *"What I will say is that we need to implement fully what has currently been allowed through the task shifting and task sharing policy"”*(Project Director), and “*To know which communities have a higher number of potential users so that you are maximizing every effort at driving demand and providing the services"* (Executive Director), which emphasizes the importance of fully implementing the task shifting and task sharing policy” (Executive Director), and the need for targeted implementation of the task shifting and task sharing policy based on an understanding of the communities with a higher demand for DMPA-SC services. Furthermore, statements such as *"A lot of partners have been working in different subnational levels to ensure that these policies are implemented and that more providers at the lower cadre can be trained to provide the services"* (Project Director), and *"Training, appropriate training, and allocating enough time for that thing is important"* (Technical Lead), highlights the collaborative efforts of various partners in different subnational levels to support the implementation of the task shifting and task sharing policy, and underscores the significance of comprehensive and appropriate training for healthcare providers involved in the implementation of the task shifting and task sharing policy.

#### Synchronise advocacy and policy

The study highlights the importance of advocacy and policy synergy in addressing policy gaps in family planning and DMPA-SC programme implementation. It emphasizes the need for resource allocation, engagement with key stakeholders, and the adoption the Task Shifting and Task Sharing policy and the Patent and Proprietary Medicine Vendors (PPMV) tier accreditation policy by the government at all levels. Advocacy has also been identified as playing a crucial role in convincing policymakers and the government to prioritize and adequately fund family planning initiatives. Findings identified advocacy and policy synergy as a major theme in addressing policy gaps. Common statements such as *"All we need to do now is to provide resources, engage the right personnel, engage the right leaders in the communities, in the mosque and in the churches so that the messages will be imbibed"* (Project Director), and *"Majorly, it's by advocacy. To really convince our policymakers and the government to fund family planning appropriately, release the money on time, and release all the money allocated to family planning"* (Project Director), points to the importance of resource allocation and engaging key stakeholders, including community leaders, religious leaders, and personnel, in advocating for family planning, and further highlights the critical role of advocacy in influencing policymakers and the government to prioritize and adequately fund family planning initiatives. Furthermore, statements such as *"In the policy space, creating an enabling environment, we want to see some synergy between the primary healthcare development agency and the private sector"* (M&E officer), and *"But that should be the TSTS policy and the PPMV tier accreditation policy should be adopted widely by the government"* (Project Director), emphasizes the importance of creating an enabling environment for family planning through policy synergy between the primary healthcare development agency and the private sector and points to the importance of adopting policies such as the task shifting and task sharing (TSTS) policy and the patent and proprietary medicine vendors (PPMV) tier accreditation policy by the government.

#### Review and revise policies

Policy revision is a key strategy to addressing the policy gaps in family planning implementation in Nigeria. Focusing on reviewing existing policy documents related to the introduction and scale-up of DMPA-SC by ensuring they align with the objectives and strategies of its implementation process is important. Results from responses revealed policy review and revision as a major theme in addressing policy gaps. The following statements point to this: *"It is also instructive that it is time to review some of them, so we will be looking at all the documents and trying to connect the dots so that we don't have these documents in silos"* (Rep Pharmaceutical Society of Nigeria), which highlights the need for reviewing existing policy documents related to family planning, including those pertaining to the introduction and scale-up of DMPA-SC. And *"So, that is another thing we are trying to do because that is what really guides the implementation process, so we need to be sure that the document fits"* (Rep Pharmaceutical Society of Nigeria), which emphasizes the significance of policy documents in guiding the implementation of family planning programs, including the scale-up of DMPA-SC. It underscores the need to ensure that the policy document aligns with the objectives and strategies of the implementation process. Also, the statement *"The policy document supporting its introduction and scale-up has expired, it needs to be revised, and that could give us the opportunity to bring in some imagery, role and out for a national consensus and such make them get their way to the policy"* (Clinical Director), highlights the expiration of the policy document related to the introduction and scale-up of DMPA-SC. It emphasizes the need for revising and updating this policy document to align with current needs and opportunities.

#### Strengthen referral systems and community mobilisation

Strengthening referral systems and community mobilization are identified strategies for addressing policy gaps in the Nigerian healthcare system. The importance of a strong referral system allowing healthcare providers to refer patients to appropriate facilities for further evaluation or management was brought to light. Collaboration between primary healthcare providers and government agencies is also highlighted to ensure successful integration and utilization of DMPA-SC within the broader method mix of family planning services. Community-based mobilization has also been emphasized as veritable means of disseminating information and raising awareness about DMPA-SC, particularly in areas where direct programme implementation partner engagement may be limited. The following common responses attests to this: *"If there's a complication, I should be able to refer, whether referring for the private sector or public sector or whatever, I should be able to refer"* (Rep. Pharmaceutical Society of Nigeria), and *"So, the referral system is what we want to see strengthened"* (Rep. Pharmaceutical Society of Nigeria), which emphasizes the importance of a strong referral system within the healthcare system, and the need for healthcare providers to have the capability to refer patients, including those using DMPA-SC, to appropriate facilities or providers for further evaluation or management in case of complications or specialized care. And also underscores the focus on enhancing the referral system as part of efforts to address the policy gap. Further statements such as *"Working with PHC and other government agencies to ensure that we don't have any issues, so that DMPA-SC can thrive within the method mix"* (Rep Pharmaceutical Society of Nigeria), highlight the collaboration between primary healthcare (PHC) providers and other government agencies to ensure the successful integration and utilization of DMPA-SC within the broader method mix of family planning services. And *"Where partners could not reach out, their community-based mobilization, the chips are this information available to them as well, they have the right information, can they go to the community and spread this message for DMPA-SC"* (Family Planning Manager), points to the importance of community-based mobilization in disseminating information and raising awareness about DMPA-SC. It recognizes the role of partners and community health workers in reaching out to communities, particularly in areas where direct partner engagement may be limited.

#### Advocate to policymakers

Advocacy to policymakers is crucial for addressing policy gaps in Nigeria's DMPA-SC implementation. The advocacy efforts of non-governmental organizations and community groups have brought attention to the policy gap in DMPA-SC implementation and have driven positive changes. Additionally, targeted campaigns have been suggested as veritable tools for raising awareness and advocating for appropriate actions and resource allocation for DMPA-SC implementation. The following statements point to this: *"But I know that advocacies have been going on in terms of SP Nigeria, but things are improving"* (Project Director), and *"Yeah, I mean it's simple advocacy to relevant policymakers"* (Clinical Director). These statements acknowledge the importance of the ongoing advocacy efforts of non-governmental organizations and advocacy groups working in the field of reproductive health and also suggest that these advocacy initiatives have been instrumental in bringing attention to the policy gap and driving positive changes. This also emphasises the importance of conducting advocacy campaigns directed at relevant policymakers; that by engaging policymakers through targeted advocacy efforts, it is possible to raise awareness about the policy gap and advocate for appropriate actions and resource allocation.

### Recommendations for improving implementation

Recommendations for improving the provider-administered and self-injection DMPA-SC programme implementation are captured under the following themes;

#### Strengthen service provider capacity

The importance of strengthening service providers’ capacity to deliver DMPA-SC services in line with global best practices has been emphasized. This can be achieved through training, both formal and on-the-job, and by ensuring that health institutions'curricula incorporate family planning contents, including DMPA-SC. This will equip future health workers with the knowledge and skills to provide family planning services. Continuous improvement in the capacity of service providers to deliver DMPA-SC services in line with the global best practices through training was brought to bear from common responses as a veritable tool for improving provider-administered and self-injection DMPA-SC services as well as for other family planning services. *"Strengthen capacity in health services and procure commodities"* (Programme Manager), and"*Building the capacity of more health workers and supporting health institutions to train them"* (Project Director). This further highlights the importance of strengthening the capacity of health services to provide family planning services. This can be done by training health workers in formal settings and through on-the-job training. Also, the statement *"Ensure the curricula for health institutions incorporate family planning, including DMPA-SC"* (Project Director). This highlights the importance of ensuring that family planning is included in the curricula of health institutions producing low and middle-cadre healthcare care workers. This will help to ensure that future health workers are equipped with the knowledge and skills to provide family planning services.

#### Digitize data collection system and monitoring systems

The need for digitizing data collection and monitoring systems for DMPA-SC programme implementation will improve data collection and monitoring systems for proper accountability. The participants expressed this view: *"Digitize data collection processes for efficient monitoring"* (State Reproductive Health Coordinator), and *"Establish a prescribed data collection system for DMPASC"* (Clinical Director), these statements highlight the importance of digitizing data collection processes in order to improve efficiency and monitoring; and emphasises the need to establish a prescribed data collection system for DMPA-SC.

#### Deploy innovative financing and domestic resourcing system

Innovative financing and domestic resourcing systems were recommended drivers of improved DMPA-SC programme implementation. The following statements attest to this:"*Innovate financing mechanisms and mobilize more domestic resources"* (Family Planning Manager), and *"Invest in policies related to DMPASC and revise family planning methods"* (Male Clinical Director), which highlights the importance of innovating financing mechanisms and mobilizing more domestic resources to support family planning programs; and emphasizes the need to invest in policies related to DMPA-SC and revise family planning methods. Also, the statement “*Ensure regular review of policies and capture the magnitude of family planning needs…”* (Clinical Director), highlights the importance of ensuring regular review of policies and capturing the magnitude of the unmet need for family planning.

#### Strengthen coordination mechanism

Enhancement of a strong coordination mechanisms and promotion of engagement between the public and private sectors is opined to improved utilization of DMPA-SC and this was alluded to by the following responses. *"Strengthen coordination mechanisms and public–private sector engagement"* (Programme Manager), emphasizes the need to promote engagement between the public and private sectors for improved programme implementation. And the statement *"Improve private sector engagement and sustainable tenants"* (Family Planning Manager), point to the importance of engaging the private sector and fostering sustainable partnerships. While the statements: *"Ensure better coordination and collaboration between government and partners”* (Female State Reproductive Health Coordinator), and *"Promote regular stakeholder engagement and community engagement"* (Project Director), underscores the need for enhanced coordination and collaboration between the government and partner organizations involved in family planning programming; and highlights the significance of regular engagement with stakeholders and the community in family planning programs.

#### Improve funding investment

Addressing the funding gap and exploring alternative mechanisms for resource mobilization is essential for improving the sustainability of DMPA-SC and family planning programming. Hence, there is a necessity for a financial handshake between the federal and state governments, as well as partner organizations, to ensure uninterrupted family planning programming, including DMPA-SC. This is evident in the responses obtained from the stakeholders. “*Bridge the national funding gap and alternative resource organization mechanisms is essential"* (Programme Manager), and *"Mobilize funds from federal and state governments and partners"* (Programme Director), *"Ensure sufficient supply of commodities and availability of resources for service provision”,"Mobilize more domestic resources to support DMPA-SC interventions"* (Program Officer).

#### Review DMPA-SC policies and evaluate programme periodically

The need to review and revise policies related to DMPA-SC and other family planning methods emphasizes the importance of ensuring that they are comprehensive, up-to-date, and relevant to DMPA-SC service delivery, as highlighted by these statements. *"Revise policies related to DMPA-SC and other family planning methods"* (Clinical Director), and *"Invest in policies related to DMPASC and revise family planning methods"*. (Clinical Director).

#### Build awareness and generate demand continuously

Building awareness and demand generation is pivotal to improving the DMPA-SC programme implementation and scale-up in Nigeria. The following statements attest to this: *"Conduct demand generation activities and create awareness at the community level"* (Director of Pharmaceutical Services), and *"Ensure adequate awareness creation and counselling for acceptance of self-injection"* emphasizes the importance of conducting demand generation activities and creating awareness about DMPA-SC at the community level; and highlights the need to ensure adequate awareness creation and counselling specifically for the acceptance and uptake of self-injection of DMPA-SC. Also, the statement *"Continue to make DMPA-SC available, avoid stockouts, and ensure reporting and provider availability"* (Programme Coordinator), emphasizes the importance of ensuring the continued availability of DMPA-SC, avoiding stockouts, and ensuring reporting and provider availability.

#### Apply evidence-based approach to learning

Evidence-driven action is essential for improved DMPA-SC and family planning programme implementation outcomes. This is attested to by responses elicited from the participants as *"Designing programs based on evidence is the way to go"* (Programme Director), which emphasizes the importance of adopting an evidence-based approach in designing programs related to the introduction of DMPA-SC and the statement *"Generating learning and inputting it into the system, along with best practices, is crucial"* (Clinical Director), which highlights the value of generating and incorporating learning into the healthcare system during the introduction of DMPA-SC. Also, the statement *"Implementation science and the concept of each community are important lessons learned"* (Clinical Director), recognizes the importance of implementation science and community-specific approaches in introducing DMPA-SC. And the statement, *"Evidence is necessary to understand barriers, tailor communication strategies, monitor implementation, and make necessary corrections"* (Chief Executive Officer). This underscores the critical role of evidence-driven action in identifying barriers to access and utilization, developing communication strategies and monitoring programme implementation progress.

## Discussion

DMPA-SC is a new, simple-to-use injectable contraception that is suited for self-administration [24]. The introduction of provider-administered and self-injection DMPA-SC is one of the strategies employed by the Federal government of Nigeria’s national family planning goals to meet the needs for contraception, especially in hard-to-reach communities. Provider-administered and self-injection DMPA-SC have demonstrated the potential to achieve the desired scale-up and reach if given the needed priorities and enablement [25]. This assessment revealed improved DMPA-SC uptake and acceptance over the years. This success was observed to have been supported by the existence of functional policies and guidelines, while scale-up was impeded by funding gaps, human resource constraints, and weak coordination mechanisms. In furtherance, the continuous implementation of the TSTS policy and the regular review and update of other related policies and guidelines, were suggested actions that could strengthen the implementation and scale-up of the DMPA-SC programme in the country. Additionally, strengthening of the referral systems and decentralisation of family planning services were also expressed as notable and home-grown actions that could positively influence DMPA-SC implementation in Nigeria.

Other studies conducted in sub-Saharan African countries by FHI 360, PATH, and other partners have generated empirical evidence showing that provider-administered and self-injection DMPA-SC uptake is increasing [19, 25, 26]. This may be because it promotes privacy and offers women control over their reproductive choices, in addition to other economic benefits [27–30]. Furthermore, the Nigerian government started the implementation of the TSTS policy in the family planning space, which allows the trained community service providers to administer and show women how to self-inject DMPA-SC which in a way could have also contributed to increase in acceptance and uptake of the DMPA-SC [31]. The implementation of the TSTS policy as facilitator for improvement in provider-administered and self-injection DMPA-SC uptake was also alluded to by findings other studies [31–34]^.^ This has further reiterated the importance of enabling policy environment as nexus for seamless programme implementation. The implementation gaps in provider-administered and self-injection DMPA-SC identified in this assessment were inadequate funding, distribution and logistics challenges, human resources and capacity deficits, and weak coordination mechanisms. Other assessments and studies also found similar barriers, such as sub-optimal funding, inadequacy of the critical pool of trained health personnel, and poor logistics and supply chain systems [34–36]. These impediments appear to cut across climes and regions, and their repeated emergence indicates the need for immediate and concerted efforts to overcome them. Given the identified barriers to provider-administered and self-injection DMPA-SC programme implementation, continued implementation of TSTS policy, digitization of the data collection system, improved advocacy and demand generation, as well as strengthening of synergy in family planning policies are prescribed innovative ways for promoting DMPA-SC programme implementation in Nigeria. Other studies make recommendations similar to that of this assessment, which include task-sharing amongst health workforce cadres; engaged leadership; strong and enforced policies; training and education; demand generation; integration of provider-administered and self-injection DMPA-SC into existing programs; improved funding mechanisms; collaboration with development partners; and supply chain strengthening [37–42]. It is sufficient to state that if the DMPA-SC programme implementation is hinged on these programme levellers, then continuous scale-up and sustainability are achievable. Additionally, combining provider-administered and self-injection DMPA-SC services with proactive community-based distribution has been identified as another veritable strategy for accelerating DMPA-SC uptake [41, 42]. This implies that successful implementation of DMPA-SC programme will require concerted efforts of all stakeholders at both supply and demand sides.

### Limitations

It is important to state that the findings of this assessment are limited to the stakeholders’ perspectives in the supply side of the DMPA-SC programme implementation such that generalizing to the full complement of the DMPA-SC programme implementation (Supply and Demand sides) is not feasible. Hence it will be imperative that further studies be conducted to wholistically explore both the demand and supply sides as a way of gaining the perspectives of the end users and private sector as well to have a full picture of the DMPA-SC implementation in Nigeria.

### Recommnedations

To strengthen the implementation and scale-up of the DMPA-SC programme in Nigeria, training of service providers, improved funding through the use of innovative financing, continuous implementation of the TSTS policy, regular review and update of related family planning policies and guidelines and strengthening of referral systems are recommended levers.

## Conclusion

This assessment shows an upward trend in provider-administered and self-injection DMPA-SC uptake and acceptability in Nigeria, facilitated by policy enforcement and committed government and partners. Service provider training and capacity building; periodic policy review; data collection and monitoring digitalisation; and improved funding through innovative financing were recommended to scale up and sustain provider-administered and self-injection DMPA-SC.

## Data Availability

The datasets used and/or analysed during this study are available from the corresponding author on reasonable request.

## Abbreviations

AC: Access Collaborative
DMPA-SC: Subcutaneous depot-medroxyprogesterone acetate
IUD: Intrauterine device
KII: Key informant interview
LGA: Local government area
NAFDAC: National Agency for Food and Drug Administration and Control
SI: Self Injection
TSTS: Task-shifting and task-sharing
